# Validity and Reliability of Self-Assessment Tool for Risk Prioritization Following Exposure to Tuberculosis in a Hospital Setting

**DOI:** 10.3390/ijerph18083981

**Published:** 2021-04-09

**Authors:** Piyapong Sirinapakul, Naesinee Chaiear, Phanumas Krisorn

**Affiliations:** 1 Division of Occupational Medicine, Department of Community Medicine, Faculty of Medicine, Khon Kaen University, Khon Kaen 40002, Thailand; pisirin@kkumail.com (P.S.); phankr@kku.ac.th (P.K.); 2 Occupational Health and Safety Office, Faculty of Medicine, Khon Kaen University, Khon Kaen 40002, Thailand

**Keywords:** tuberculosis, health worker, validity, reliability, surveillance

## Abstract

The Modified Self-Assessment (MSA) and Present Self-Assessment (PSA) forms are questionnaires used to prioritize the risk of infection of health workers exposed to tuberculosis (TB) in Srinagarind Hospital in Thailand. As MSA was developed from PSA, the validity and reliability of MSA need to be assessed. The research aim is to examine the content validity of MSA and to assess the respective reliability of MSA and PSA vis-à-vis expert opinion. Seven experts determined the content validity index (CVI) of MSA. MSA and PSA were used to prioritize the TB contact of 108 subjects, and we compared the result with the risk assessed by the experts. The respective Kappa agreements between MSA and PSA and the experts were used to assess reliability. The result of the content validity index revealed that MSA had I-CVI > 0.83 for all questions and an S-CVI/Ave above 0.90 for all factors. The Kappa agreement of contact priority between MSA and the experts was 0.80; it was 0.58 between PSA and the experts. MSA can, thus, be used to prioritize contact with tuberculosis in Srinagarind Hospital. MSA is a valid risk communication tool for aerosol-generating procedures. Further study should be conducted in other hospitals, and the number of participants should be increased in order to come to a concrete result.

## 1. Introduction

Tuberculosis (TB) infection is considered an urgent global health issue [1]. The World Health Organization (WHO) has devised a three-pronged strategy to end TB globally: (1) Reach 90% of all people who need TB treatment, (2) include 90% of all people in key populations, and (3) achieve at least 90% treatment success [2]. Health workers (HWs) are a key population because they have a 2–4 times increased risk of TB infection compared to the general population [3]. Occupational latent TB infection (LTBI) can lead to active TB (aTB), which can be a source of transmission to others and a complicated infection in and of itself [4,5]. LTBI screening and LTBI management in HWs are necessary in order to achieve WHO’s “End TB Strategy” [6]. 

There are three screening models for LTBI surveillance in HWs: preplacement screening, periodic screening, and postexposure screening. The Center of Disease Control and Prevention (CDC) recommends all HWs should be screened for LTBI (i.e., preplacement and postexposure) [7]. Postexposure screening and treatment of LTBI should be considered a strong recommendation for screening HWs for LTBI [6,7,8,9]. 

Thailand has a high burden of TB; thus, HWs encounter complicated cases of TB with comorbidities. As a consequence, TB infection among HWs is often due to occupational exposure [3,10]. Contact investigations play an integral role in the strategy for ending TB, and these activities were introduced in the 2005 CDC Guideline and the 2007 Maryland Guideline [11,12]. These guidelines are the protocol of contact investigations, involving complex decisions with respect to the investigation priority of HWs after exposure to TB using epidemiology triad components: hosts (HWs), agents (TB patients), and environment (contact activity, contact duration, and room size).

The Occupational Health and Safety Office (OH&S) at the Faculty of Medicine, Khon Kaen University Thailand (KKU) created a Present Self-Assessment (PSA) form—a tool for prioritizing contact investigations after occupational TB exposure [10]. The PSA content was adopted from the two guidelines mentioned [11,12]. HWs exposed to TB have their contact prioritized using PSA. High and medium priority contact are followed up for LTBI/aTB investigation. Panthong et al. [10] studied the benefit of prioritization using PSA and found that the results were not consistent with the interferon-gamma release assay test. HWs’ confusion regarding some of the PSA questions led to inappropriate contact prioritization. The validity and reliability of PSA have not been assessed as some PSA content that is essential to determining contact priority is lacking. The present study aims to (1) examine the content validity of the Modified Self-Assessment (MSA) form and (2) assess the respective reliability of MSA and PSA with respect to expert opinion.

## 2. Materials and Methods

### 2.1. Study Design

A three-step descriptive study was conducted. First, the MSA form was created by improving the content of the PSA form. Second, MSA was examined for content validity by seven experts. Third, its reliability was assessed using agreement measurements between both PSA and MSA and the experts.

### 2.2. Study Population and Sample

#### 2.2.1. Study Population for Examining Content Validity 

Seven eligible content validity experts were asked to examine the content validity of MSA. The seven included 1 chest medicine doctor, 3 occupational medicine doctors, 1 infectious control manager, 1 safety officer, and 1 epidemiologist. 

#### 2.2.2. The Study Population of Reliability Assessment

The reliability expert was an occupational medicine doctor with two years of experience in contact TB history prioritization using the two guidelines. The reliability expert prioritized the TB contact of subjects with regard to their TB contact history through telephone interviews.

Srinagarind Hospital has a TB contact investigation procedure. All HWs are to report the contact event to OH&S in order to follow the procedure. HWs who reported the contact event between 1 January 2020 and 31 March 2020 were asked to participate in this study. The sample size (*n* = 108) was calculated using Cohen’s kappa sample size calculation using the following formula [13]:

$$n=[(Z_{\alpha }\sqrt{Q_{0}}\;+\;Z_{\beta }\sqrt{Q_{1}})/(K_{1}-\;K_{0}){]}^{2}$$

where *n* = sample size

*Z_α_* = 95% CI = 1.96

*Z_β_* = 80% of power testing = 0.84

*K*_0_ = lower border of Kappa = 0.60

*Q*_0_ = coefficient of K_0_ = 0.64

*K*_1_ = upper border of Kappa = 0.80

*Q*_1_ = coefficient of K_1_ = 0.36

One hundred and eight subjects, who had given informed consent, were asked to complete both MSA and PSA within 72 h after contact. They also provided their contact history to the reliability expert through a telephone interview within 5 days of completing MSA and PSA. 

### 2.3. MSA and PSA

Both MSA and PSA comprise questions relating to the aTB and LTBI risk factors: (1) infectivity of the index case, (2) personal factors of the HWs (underlying disease, health status, and medication), (3) contact activity, and (4) environmental factors (contact duration, size of the room). MSA represents an improvement on PSA as it has more comprehensive questions regarding these factors [14,15,16,17,18]. MSA and PSA are included in Table A1 and Table A2 of Appendix A.

**Figure 1:**
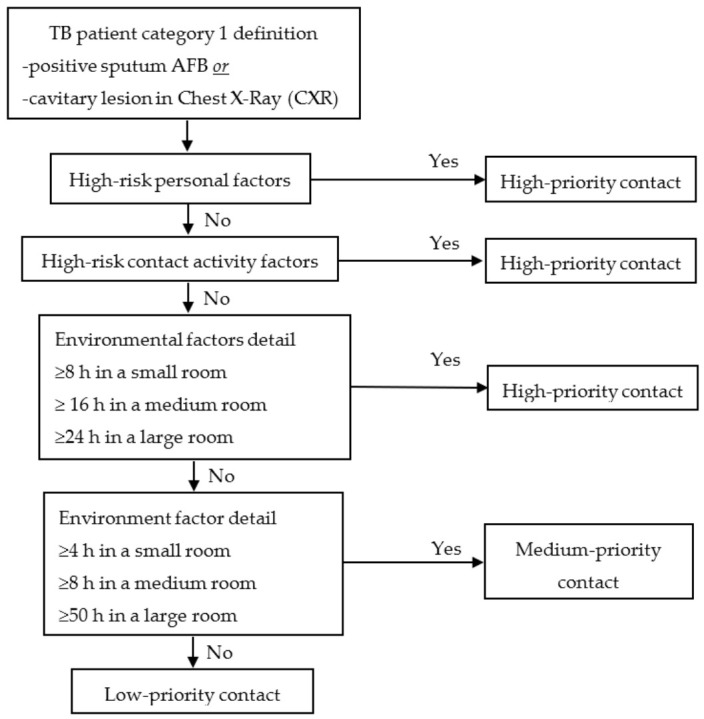
Prioritization of contacts exposed to tuberculosis (TB) Patient Category 1. (Source: OH&S Faculty of Medicine KKU Thailand).

**Figure 2:**
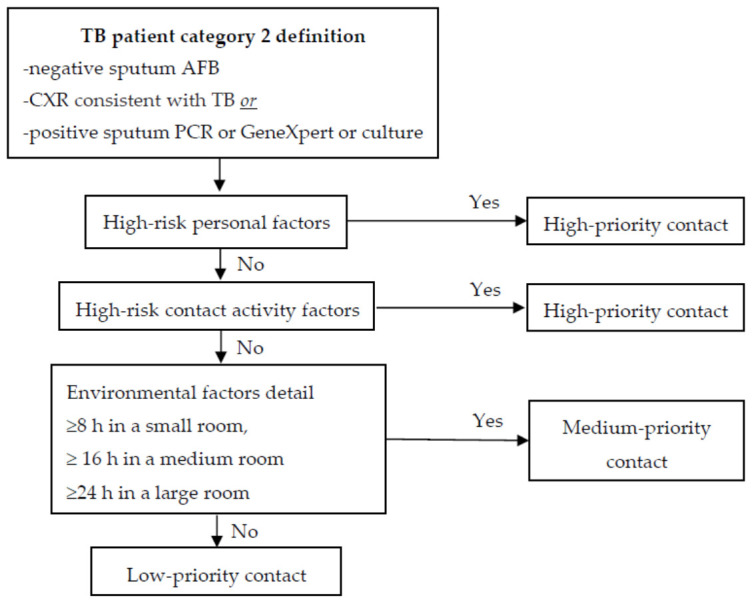
Prioritization of contacts exposed to TB Patient Category 2. (Source: OH&S Faculty of Medicine KKU Thailand).

**Figure 3:**
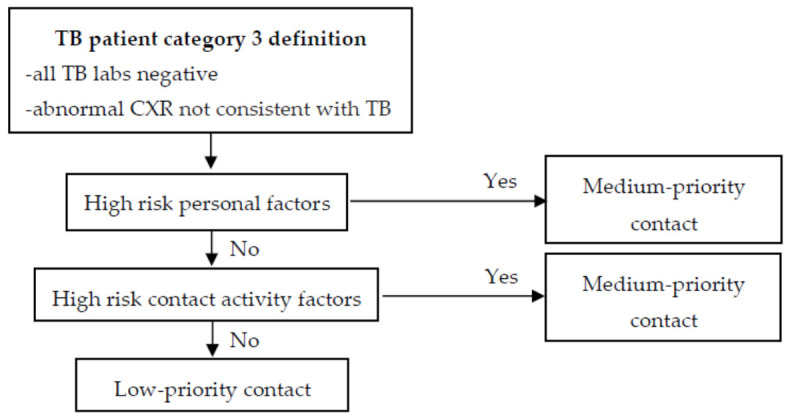
Prioritization of contacts exposed to TB Patient Category 3. (Source: OH&S Faculty of Medicine KKU Thailand).

A TB patient will be categorized into one of three levels based on infectivity. When HWs have contact with each TB patient category, the priority of contact is determined step-by-step: personal factors (risk of aTB), contact activity (risk of LTBI), and environmental factors (risk of LTBI). The steps of the contact prioritization process are presented in Figure 1, Figure 2 and Figure 3, according to the category type [10].

### 2.4. Study Tool

A content validity examination form (CVEF; shown in Table A3 of Appendix A) was used by seven experts. Each question of CVEF is rated on a 4-scale (strongly agree, agree, disagree, and strongly disagree). For reliability assessment, a data record sheet was completed by the reliability expert. The contact history collected during the telephone interviews with each of the 108 subjects was recorded on 108 separate datasheets. 

### 2.5. Analysis

#### 2.5.1. Content Validity of MSA

Since the data of MSA were facts, the content validity examination was proper. We used the Item Content Validity Index (I-CVI) and the Scale Content Validity Index per Average (S-CVI/Ave) for data analysis of content validity. I-CVI was calculated from the proportion of experts who “strongly agreed” or “agreed” to each question item of CVEF. S-CVI/Ave refers to the content validity of the factor, calculated from the average value of all I-CVI scores. The cutoff values of I-CVI and S-CVI/Ave depend on the number of experts. Polit and Beck [19] suggested the acceptant of I-CVI and S-CVI/Ave, in terms of 6–8 experts, should ≥0.83 and ≥0.90, respectively. The I-CVI and S-CVI/Ave formulas are presented in Table A3 of Appendix A. 

#### 2.5.2. Reliability of MSA and PSA

Reliability was assessed using Cohen’s kappa (Kappa) agreement between MSA and expert opinion and PSA and expert opinion using the following formula:

*K* = (*P*_0_ − *P_E_*)/(1 − *P_E_*)

where

*P*_0_ = proportion of observed agreement

*P_E_* = proportion of expected agreement

IBM SPSS Statistics version 19 (with Khon Kaen university’s license) was used for Kappa analysis; the process of calculation is demonstrated in Table A4 and Table A5 of Appendix A. An acceptable Kappa value for a moderate level of reliability is considered to be ≥0.60 [20]. Kappa agreement analysis is a critical aspect in determining contact priority. There are three levels of contact priority—high, medium, and low. Personal factors, contact activity factors, and environmental factors were also included in the Kappa agreement analysis, albeit these have only minor agreement. 

Personal factors have two levels of priority, the same as the contact activity factors. The level is defined as high risk and low risk. For example, HWs with items in personal factors are marked as high-risk for aTB; HWs who performed “suction” (a contact activity factor) are marked as high-risk for LTBI. On the other hand, HWs who are healthy or have other conditions that are not present in personal factors are marked as low-risk for aTB; HWs who perform “oral medication” (a contact activity factor) with TB patients are marked as low-risk for LTBI. 

As for environmental factors, the duration of contact has 6 levels of priority, including ≤4, 4–8, 8–16, 16–24, 24–50, and ≥50 h. Room size has three levels of priority—small, medium, and large rooms.

Kappa agreement was calculated based on the comparison between each level of priority between MSA and expert opinion and PSA and expert opinion.

### 2.6. Ethics

The primary ethical concern was the confidentiality of personal data. This report was certified by the Institutional Review Board (Number IRB00001189) and the Khon Kaen University Research Ethics Committee (HE621446).

## 3. Results

### 3.1. Demographic Data

The 108 subjects who participated in the reliability study had been exposed to TB patients. The subjects comprised nurses (50.9%), nurse assistants (20.4%), and technicians (14.8%). The contact workplace included inpatient departments (45%), intensive care units (23%), and laboratory units (19%). The demographic data are shown in Table 1.

### 3.2. Content Validity Assessment of MSA

As for personal factors, the items that had an I-CVI value of 0.86 were “bronchiectasis” and “BMI < 18.50 kg/m^2^” because one of seven experts evaluated these items as low-risk for aTB. By contrast, the rest of the personal factors had an I-ICVI value of 1 as all seven experts agreed that these carried a high risk of aTB. The S-CVI/Ave value for personal factors was 0.99.

The contact activity factor for MSA was divided into high risk for LTBI and low risk for LTBI. As to high risk, the items had an I-CVI value of 0.86, including “autopsy” and “carry sputum”. As to low risk, the items had an I-CVI value of 0.86, including “cleaning the room”, “bathing in bed”, “transferring the patient”, “vital sign assessment”, and “talking to the patient”. Thus, the S-CVI/Ave value for contact activity factors was 0.96.

Room size is part of the environmental factors. The priority levels for room size are small, medium, and large. The I-CVI value for a small room, which includes examination rooms, individual rooms, operative rooms, bronchoscopy rooms, and X-ray rooms, was 0.86. The I-CVI value for medium rooms, which include wards, emergency rooms, OPD stations, and recovery rooms, was 0.86. The remaining items, vis-à-vis room size, had an I-CVI value of 1 because the seven experts strongly agreed/agreed with the content. Thus, the S-CVI/Ave value for environment factors was 0.91. The resulting content validity assessment is presented in Table 2.

### 3.3. Reliability Assessment of MSA and PSA

The Kappa agreement for contact priority between MSA and the experts was 0.80 (95% CI: 0.70, 0.90) vs. 0.58 (95% CI: 0.44, 0.72) between PSA and the experts. The Kappa agreement for (1) personal factors regarding (a) underlying diseases between MSA and the experts was 0.87 (95% CI: 0.77, 0.96) and between PSA and the experts, 0.59 (95% CI: 0.43, 0.76), (b) health status between MSA and the experts was 0.64 (95% CI: 0.37, 0.91), and (c) medication between MSA and the experts was 1; (2) contact activity factors between MSA and the experts was 0.75 (95% CI: 0.62, 0.88) and between PSA and the experts, 0.84 (95% CI: 0.73, 0.95); (3) environment factors regarding (a) contact duration between MSA and the experts was 0.50 (95% CI: 0.36, 0.64) and between PSA and the experts, 0.23 (95% CI: 0.13, 0.33), and (b) room size between MSA and the experts was 0.70 (95% CI: 0.59, 0.81) and between PSA and the experts, 0.56 (95% CI: 0.43, 0.69). The Kappa agreement for contact priority and factors are presented in Table 3 and Table 4, respectively.

## 4. Discussion

The purpose of the current study is to develop the Modified Self-Assessment form, a tool for prioritizing HWs who have been in contact with TB patients. The MSA content was improved from PSA by adding items to the personal factors, contact activity factors, and environmental factors categories, which are risks for aTB and LTBI [14,15,16,17,18]. Content validity was then re-examined. The reliability of MSA was assessed by determining the Kappa agreement between MSA and the experts. Additionally, the reliability of PSA was assessed in order to compare MSA and PSA.

### 4.1. Validity Assessment

The content validity of MSA passed the I-CVI and S-CVI/Ave criteria. To pass the I-CVI criteria (≥0.83), an item should have agreement from at least 6 experts, resulting in an I-CVI value of 0.86. The items in the three factors of MSA comprise both clinical and epidemiological content. Seven experts were selected because they were clinicians (a chest specialist, three occupational medicine specialists, and three epidemiologists). The differences in expert subject areas might account for disagreements on some items. For example, regarding contact activity factors, one of the epidemiologists disagreed that “bathing in bed” and “talking to the patient” were low-risk for LTBI because HWs might come into contact with TB through aerosol droplets in the air or on surfaces during these activities. By contrast, clinicians suggested that these items were low-risk because they are not aerosol-generating procedures (AGPs). 

The literature rates the most common risks for aTB as those related to personal factors [14,15,16,17,18]. As such, the resulting content validity confirmed that personal factors had the highest number of single points of I-CVI, contributing to the highest S-CVI/Ave. On the other hand, according to the experts, contact activity factors and environmental factors had a lower S-CVI/Ave value than personal factors.

### 4.2. Reliability Assessment

#### 4.2.1. Discussion of Contact Priority Results

We conducted a reliability assessment of the application of MSA to HWs who have come in contact with TB patients. Agreement between MSA and the experts was assessed because MSA represents the experts who make decisions on contact priority based on the contact history of the subjects. MSA had strong agreement with the contact priority of the experts (Kappa 0.80 (95% CI: 0.70, 0.90)). MSA could, thus, be applied to prioritize HWs’ contact exposure to TB. By contrast, the agreement between PSA and the experts was weak (Kappa 0.58 (95% CI: 0.44, 0.72))—PSA should not, therefore, be used to prioritize contact [20].

#### 4.2.2. Discussion of Personal Factor Results

Personal factors can also be used to evaluate the fitness for work of HWs who have come into contact with TB. The respective Kappa agreement for personal factors for MSA was 0.87 (95% CI: 0.77, 0.96), 0.64 (95% CI: 0.37, 0.91), and 1 for underlying diseases, health status, and medication. The results reveal that personal factors have a high Kappa agreement because these are the subject’s individual data, which would not be affected by recall bias. The Kappa agreement for underlying disease for MSA was higher than that for PSA (Kappa 0.87 (95% CI: 0.77, 0.96) vs. Kappa 0.59 (95% CI: 0.43, 0.76)) because MSA has more items covering personal factors than PSA. While PSA could not detect a subject at risk of aTB, MSA and the experts could. Moreover, PSA did not include items for health status and medication in the personal factors category, while MSA did.

#### 4.2.3. Discussion of Contact Activity Factor Results

Contact activity results in LTBI. AGPs play a major role in respiratory infection [21,22]. Chanpho et al. [23] postulated that the HWs who perform AGPs had a significant association with LTBI (OR = 2.04, with 95% CI: 1.20, 3.48, *p* = 0.007). Although the result of contact activity factors had a Kappa agreement for MSA than PSA (Kappa 0.75 vs. Kappa 0.84), both MSA and PSA passed the agreement criteria (Kappa ≥ 0.60). PSA has five contact activity items (i.e., bronchoscopy, ET-tube intubation, suction, CPR, and sputum induction), while MSA has 22. The most common contact activity in the current study was suction, which was present in both MSA and PSA. Agreement in MSA would be raised if the number of subjects was to be increased. Based on the expert interviews, it was found that most of the subjects were not aware of the risk of LTBI when performing AGPs. It was also noted that when subjects were completing the MSA form, the risks for communication and awareness of LTBI were confirmed as factors, whereas in PSA, they were not.

#### 4.2.4. Discussion of Environment Factor Results

Environmental factors had the lowest Kappa agreement, especially for contact duration. MSA had a weak agreement for contact duration (Kappa 0.50) and minimal agreement for PSA (Kappa 0.23). The result can be explained in two ways. First, the subjects could not recall the contact duration. Second, the six-level priority of contact duration might result in low agreement. Notwithstanding, the contact duration agreement for MSA was better than PSA due to differences between the MSA and PSA forms: subjects had to complete real contact duration for MSA, while PSA was more general and less accurate (Table A1 and Table A2 of Appendix A). 

Even though less than 12 air ventilation changes per hour is a risk for LTBI [8,24], HWs were unable to comment on ventilation during their contact activity. Studies have shown that room size can predict the risk of LTBI [12,25,26]. A room size of less than 20 m^2^ is a factor associated with LTBI for HWs. Therefore, room size is an environmental factor in the current study. As for the Kappa agreement with respect to room size, MSA was better than PSA (0.70 vs. 0.56), probably because MSA had more items on room size than PSA. 

### 4.3. Application

Job title and workplace are risk factors for TB infection. Previous studies have shown that the most commonly infected persons were nurses, physicians, nurse assistants, and medical technicians because of frequent contact with TB patients and the performance of AGPs [14,15,27,28]. The current study did not include physicians because of their recall bias. By contrast, nurses, nurse assistants, and technicians have assigned workplaces and could recall their in-place contact history more clearly. Using MSA may not be appropriate for jobs that have highly variable workplaces, as with physicians.

The hierarchy of hazard control is the key to successfully controlling TB infection among HWs [4]. Reduction of sources of infection is the best way to control exposure, for example, limiting the number of TB patients, early diagnosis, the effectiveness of TB treatment, and decreasing the duration of hospital stay [8]. AGPs should also be better controlled, i.e., by using in-line suction, site ventilation control, and warning signs. Environmental controls should be used when appropriate, e.g., room ventilation, airflow management, availability of negative pressure rooms, ultraviolet germicidal irradiation, and disinfectant [29]. Finally, personal protective equipment should be adequate for HWs. All HWs should be evaluated as fit-for-duty before working with TB patients. Additionally, they should be trained so as to have adequate knowledge for best safe work practices.

### 4.4. Limitation and Suggestion

The information from the participants might not be factual. For example, if the participants informed the experts (or MSA) of a history of diabetes mullites, this study did not test their fasting blood sugar to prove if they had diabetes mullites. This study was conducted in one hospital in Thailand. A limited number of participants might result in a wide range of 95% CI of Kappa. MSA should be tested in various hospitals with a large number of participants. Recall bias is inevitable in such a study as ours, especially vis-à-vis environmental factors. In order to reduce recall bias, environmental factors should be obtained from official hospital records instead of memory. Additionally, we should address the contact priority of MSA, as compared to the LTBI test, in order to evaluate the usefulness of MSA.

## 5. Conclusions

The MSA form has improved the content for prioritizing HWs after contact with TB. The content of MSA comprises three categories associated with LTBI and aTB (viz., personal factors, contact activity factors, and environmental factors). The content validity of MSA was affirmed by seven experts. The reliability of MSA was confirmed using the respective agreement between PSA and MSA and the experts. Agreement of contact priority between MSA and the experts was strong (Kappa = 0.80 (95% CI: 0.70, 0.90)), whereas agreement between PSA and the experts was weak (Kappa = 0.58 (95% CI: 0.44, 0.72)). MSA is, thus, a tool that can be used for prioritizing HWs after contact with TB. MSA can also be used for risk communication to increase awareness of AGPs. Additionally, MSA can be used as a prototype for TB contact priority in high-burden countries, where there is limited LTBI screening. Further studies should be conducted in the other hospitals, and the number of participants should be increased in order to come to a concrete result.

## Figures and Tables

**Table 1:** Demographic data of subjects.

Characteristic	Number (n)	Percentage
Sex		
Female	93	86.11
Male	15	13.89
Total	108	100
Job title		
Nurses	55	50.93
Nurses’ assistants	22	20.37
Medical technicians	16	14.81
Healthcare assistants	8	7.41
Technicians	5	4.63
Ward workers	1	0.93
Physiotherapists	1	0.93
Total	108	100
Contact workplace		
Inpatient department	48	44.44
Intensive care unit	25	23.15
Laboratory unit	21	19.44
Outpatient department	11	10.19
Operative unit	3	2.78
Total	108	100

**Table 2:** Content validity of the Modified Self-Assessment (MSA) form.

Factors		Priorities	Items	I-CVI	S-CVI/Ave
Personal	Underlying disease	High risk of aTB	HIV, DM, ESRD with dialysis, silicosis, COPD, cancer, SLE	1	0.99
			bronchiectasis	0.86	
	Health status	High risk of aTB	smoking, alcohol abuse, old pulmonary TB, gastrectomy, jejunoileal bypass surgery, renal transplant,	1	
			BMI < 18.5 kg/m^2^	0.86	
	Medication	High risk of aTB	corticosteroids ≥15 mg daily for >4 weeks, multiple cancer chemotherapy agents, antirejection drugs for organ transplants, tumor necrosis factor alpha antagonists	1	
Contact activity		High risk of LTBI	bronchoscopy, ET-tube intubation, suction, aerosolized medication, oxygen therapy ≥3 LPM, chest percussion, sputum collection, airway assist using ambu-bag, chest compression, sputum examination, setting a positive pressure ventilator, setting a high-frequency ventilator	1	0.96
			autopsy, carry sputum	0.86	
		Low risk of LTBI	oral medication, IV medication, venous puncture	1	
			cleaning room, bathing in bed, transferring patient, vital sign assessment, talking to patients	0.86	
Environment	Room size	Small size	isolated room, sputum examination room	1	0.91
			examination room, individual room, operation room, bronchoscopy room, X-ray room	0.86	
		Medium size	intensive care unit (ICU, SICU, CCU)	1	
			ward, emergency room, OPD station, recovery room	0.86	
		Large size	hall, large conference room	1	

**Table 3:** Kappa agreement of contact priority between MSA and the experts vs. Present Self-Assessment (PSA) and the experts.

Contact Priority	MSA	Expert	Agreement	PSA	Expert	Agreement
High	50	46	44	44	46	34
Moderate	6	6	4	7	6	2
Low	52	56	48	57	56	47
Total	108		96	108		83
Kappa (95% CI)	0.80 (95% CI: 0.70, 0.90)			0.58 (95% CI: 0.44, 0.72)		

**Table 4:** Kappa agreement of factors between MSA and the experts vs. PSA and the experts.

Factors		Priorities	MSA	Expert	Agreement	PSA	Expert	Agreement
Personal	Underlying disease	High risk of aTB	8	8	7	5	8	4
		Low risk of aTB	100	100	99	103	100	99
		Total (n)	108		106	108		103
		Kappa (95% CI)	0.87 (95% CI: 0.77, 0.96)			0.59 (95% CI: 0.43, 0.76)		
	Health status	High risk of aTB	11	7	6	non	7	-
		Low risk of aTB	97	101	96	non	101	-
		Total (n)	108		102	108		-
		Kappa (95% CI)	0.64 (95% CI: 0.37, 0.91)			-		
	Medication	High risk of aTB	1	1	1	non	1	-
		Low risk of aTB	107	107	107	non	107	-
		Total (n)	108		108	108		-
		Kappa (95% CI)	1			-		
Contact activity		High risk of LTBI	42	30	30	33	30	28
		Low risk of LTBI	66	78	66	75	78	73
		Total (n)	108		96	108		101
		Kappa (95% CI)	0.75 (95% CI: 0.62, 0.88)			0.84 (95% CI: 0.73, 0.95)		
Environment	Contact duration	≤4 h	67	72	59	49	72	45
		4–8 h	9	15	5	17	15	4
		8–16 h	16	12	8	8	12	3
		16–24 h	8	3	3	8	3	1
		24–50 h	5	1	1	16	1	0
		≥50 h	3	5	2	10	5	0
		Total (n)	108		78	108		53
		Kappa (95% CI)	0.50 (95% CI: 0.36, 0.64)			0.23 (95% CI: 0.13, 0.33)		
	Room size	Small size	42	45	39	36	45	31
		Medium size	51	43	38	51	43	33
		Large size	15	20	11	21	20	14
		Total (n)	108		88	108		78
		Kappa (95% CI)	0.70 (95% CI: 0.59, 0.81)			0.56 (95% CI: 0.43, 0.69)		

## Data Availability

https://www.researchgate.net/profile/Naesinee-Chaiear (accessed on 13 June 2019).

## Appendix A

**Table A1:** Modified Self-Assessment (MSA) form.

Subject ID…………………..(Identify Index Case: Category 1 ☐ 2 ☐ 3 ☐) Contact Prioritize………..	
Identify Personal Data	
Have you been diagnosed with at least one of the following diseases?	
Diabetes mellitus End-stage renal disease, with dialysis HIV Malignancy/cancer	Systematic lupus erythematosus Chronic obstructive pulmonary disease (COPD) Bronchiectasis Silicosis
☐ Yes ☐ No	
Have you ever had/do you have the health status of at least one of the following conditions?	
Smoking (not quit) Alcohol abuse (have not quit) Old tuberculosis Body mass index <18.5 kg/m^2^ (now)	Gastrectomy Jejunoileal bypass surgery Renal transplant
☐ Yes ☐ No	
Have you used any of the following medication within the last 1 month?	
Please identify prescription…………………………………………………………………………	
**Identify the contact activity with the TB patient and the contact duration of each activity.**	
☐ bronchoscopy……………….......……minute(s) ☐ ET-tube intubation …………..……minute(s) ☐ suction………………….…..….……minute(s) ☐ aerosolized medication……….……minute(s) ☐ oxygen therapy ≥3 LPM………..…minute(s) ☐ chest percussion…………….......……minute(s) ☐ sputum collection …………….……minute(s) ☐ airway assist using ambu-bag………minute(s) ☐ chest compression (CPR) …….....…minute(s) ☐ sputum examination…………...……minute(s) ☐ setting a positive pressure ventilator…minute(s)	☐ setting a high-frequency ventilator…minute(s) ☐ autopsy…………………..…………minute(s) ☐ carry sputum……………….………minute(s) ☐ oral medication………….…………minute(s) ☐ IV medication…………….…………minute(s) ☐ venous puncture………….……..…minute(s) ☐ cleaning the room…………….……minute(s) ☐ bathing in bed………………….……minute(s) ☐ transferring the patient….......….……minute(s) ☐ vital sign assessment……….………minute(s) ☐ talking to the patient……………minute(s)
**Identify the contact workplace**	
☐ Isolated room, ☐ Sputum examination room, ☐ Examination room, ☐ Individual room, ☐ Operation room, ☐ Bronchoscopy room, ☐ X-ray room	☐ Ward ☐ Intensive care unit (ICU, SICU, CICU, PICU) ☐ Emergency room ☐ OPD station ☐ Recovery room ☐ Hall ☐ Large conference room

**Table A2:** Present Self-Assessment (PSA) form.

Subject ID…………………..(Identify Index Case: Category 1 ☐ 2 ☐ 3 ☐) Contact Prioritize………..			
**Identify Personal Data**			
**Have you been diagnosed with at least one of the following diseases?**			
Diabetes mellitus HIV Chronic renal failure			
☐ Yes ☐ No			
**Identify the contact activity with the TB patient and the contact duration of each activity.**			
☐ bronchoscopy ☐ ET-tube intubation ☐ suction		☐ chest compression (CPR) ☐ sputum induction	
**Identify the contact duration, contact workplace**			
How long was your contact activity with the TB patient?			
☐ ≤4 h ☐ 4–8 h	☐ 8–16 h ☐ 16–24 h		☐ 24–50 h ☐ ≥50 h
**Where was the contact place?**			
☐ Operation room ☐ Bronchoscopy room ☐ Isolated room ☐ Individual room		☐ Recovery room ☐ Resuscitation room ☐ Hall ☐ Large conference room	

**Table A3:** I-CVI and S-CVI/Ave formula and calculation process.

Personal factors	**These Underlying Diseases Are a Risk of Active TB**.	**Expert Opinion**			
		**Strongly Agree**	**Agree**	**Disagree**	**Strongly Disagree**
	HIV	1 2 3 4 5 6 7			
	Diabetes mellitus (DM)	1 2 3 4 5 6 7			
	End stage renal disease (ESRD) with dialysis	1 2 3 4	5 6 7		
	Chronic obstructive pulmonary disease (COPD)	1 3 4 5	2 6 7		
	Bronchiectasis	1	2 3 4 5 7	6	
	Any malignancy/cancer	1 2 4	3 5 6 7		
	Systematic lupus erythematosus (SLE)	1 2 3 5 7	4 6		
	Silicosis	1 2 3 4 5 6 7			
	These health statuses are a risk of active TB.	Expert opinion			
		Strongly agree	Agree	Disagree	Strongly disagree
	smoking	1 2 3 7	4 5 6		
	Alcohol abuse	1	2 3 4 5 6 7		
	Old pulmonary TB	1 2 3 4 7	5 6		
	BMI < 18.5 kg/m^2^		1 2 3 4 6 7	5	
	Gastrectomy	1	2 3 4 5 6 7		
	Jejunoileal bypass surgery		1 2 3 4 5 6 7		
	Renal transplant	1 2 3	4 5 6 7		
	These medications are a risk of active TB	Expert opinion			
		Strongly agree	Agree	Disagree	Strongly disagree
	Corticosteroids ≥15 mg daily for >4 weeks	1 2 3 4 5 6 7			
	Multiple cancer chemotherapy agents	1 2 3 4 5 6 7			
	Antirejection drugs for organ transplants	1 2 3 4 5 6 7			
	Tumor necrosis factor alpha antagonists	1 2 3 4 5 6 7			
Contact activities	These contact activities are at high-risk of latent TB infection	Expert opinion			
		Strongly agree	Agree	Disagree	Strongly disagree
	Bronchoscopy	1 2 3 4 5 6 7			
	ET-tube intubation	1 2 3 4 5 6 7			
	Suction	1 2 3 4 5 6 7			
	Autopsy pulmonary TB patient		1 2 3 4 5 6	7	
	Aerosolized medication	1 3 4 7	2 5 6		
	Oxygen therapy ≥3 LPM	1 3	2 4 5 6 7		
	Chest percussion	1 2 3 4 5 6 7			
	Sputum collection		1 2 3 4 5 6 7		
	Carry sputum (bring sputum container to lab)		1 2 3 4 5 7	6	
	Airway assist using ambu-bag	1 3 4	2 5 6 7		
	Chest compression (CPR)	1 2 3 4 5 6 7			
	Sputum examination in laboratory	5 6	1 2 3 4 7		
	Setting a positive pressure ventilator	1 2 3 4 5 6 7			
	Setting a high-frequency ventilator	1 2 3 4 5 6 7			
	These contact activities are at low risk of latent TB infection	Expert opinion			
		Strongly agree	Agree	Disagree	Strongly disagree
	Oral medication	1 2 3 4 5 6 7			
	IV medication	1 2 3 4 5 6 7			
	Venous puncture	1 2 3 4 5 6 7			
	Cleaning room	1 2 4	3 5 6	7	
	Bathing patient in bed	1 2 3 4	5 7	6	
	Transferring patient	1 2 3 4	6 7	5	
	Vital sign assessment	1 2 3 4	6 7	5	
	Talking to the patient		1 2 3 4 5 6	7	
Environment factors	These rooms are of small size, which carries a high risk of LTBI.	Expert opinion			
		Strongly agree	Agree	Disagree	Strongly disagree
	Isolated room	1 2 3 4 5 6 7			
	Examination room	1 2 3 4	5 7	6	
	Individual room	1 2 3	4 5 7	6	
	Bronchoscopy room	5 6 7	1 2 3	4	
	Sputum examination room	5 6 7	1 2 3 4		
	Operation room		1 2 3 4 5 7	6	
	X-ray room		1 2 3 4 5 7	6	
	These rooms are of medium size, which carries a medium risk of LTBI	Experts opinion			
		Strongly agree	Agree	Disagree	Strongly disagree
	Intensive care unit (ICU, SICU, CCU)	1 2 3 4 5 6 7			
	Ward		1 2 3 4 5 6	7	
	Emergency room		1 2 3 4 5 6	7	
	OPD station	5 6 7	2 3 4	1	
	Recovery room	6 7	1 2 4 5	3	
	These rooms are of large size, which carries a low risk of LTBI	Experts opinion			
		Strongly agree	agree	disagree	Strongly disagree
	Hall	1 2 3 4 5 6 7			
	Large conference room	1 2 3 4 5 6 7			

Experts’ identification: 1 = chest medicine doctor; 2, 3, 4 = occupational medicine doctors; 5 = infectious control manager; 6 = safety officer; 7 = epidemiologist. Formula of I-CVI and S-CVI/Ave: I-CVI = (number of experts who “Strongly agree” or “Agree”)/(total nmber of experts). S-CVI/Ave = (total value of I-CVI in the factor)/(number of items in the factor) For example: I-CVI of the bronchiectasis item in personal factors = 6/7 = 0.86; S-CVI/Ave of personal factors = [(1 + 1 + 1 + 1 + 0.86 + 1 + 1 + 1) + (1 + 1 + 1 + 0.86 + 1 + 1 + 1) + (1 + 1+1 + 1)]/19 = 0.99.

**Table A4:** Kappa calculation formula and calculation process.

MSA	Experts	Number of Agreement (%)	PSA	Experts	Number of Agreement (%)
High	High	44 (40.7)	High	High	34 (31.5)
High	Medium	0	High	Medium	2 (1.9)
High	Low	6 (5.6)	High	Low	8 (7.4)
Medium	High	0	Medium	High	4 (3.7)
Medium	Medium	4 (3.7)	Medium	Medium	2 (1.9)
Medium	Low	2 (1.9)	Medium	Low	1 (0.9)
Low	High	2 (1.9)	Low	High	8 (7.4)
Low	Medium	2 (1.9)	Low	Medium	2 (1.9)
Low	Low	48 (44.4)	Low	Low	47 (43.5)
Total agreement		96 (88.9)	Total agreement		83 (76.8)
Kappa (95% CI)		0.80 (95% CI: 0.70, 0.90)	Kappa (95% CI)		0.58 (95% CI: 0.44, 0.72)

*K* = (*P*_0_ − *P_E_*)/(1 − *P_E_*); *SD(K)* =$\;\sqrt{\frac{P_{0}\left(1-P_{0}\right)}{{\left(1-P_{E}\right)}^{2}}}$; where *P*_0_ = proportion of observed agreement; *P_E_* = proportion of expected agreement. The raw data of contact priority between MSA and the experts and PSA and the experts.

**Table A5:** Calculation of agreement of contact priority between MSA and the experts.

		Expert			T_I_
		High	Medium	Low	
**MSA**	**High**	44	0	6	50
	**Medium**	0	4	2	8
	**Low**	2	2	48	52
**U_i_**		46	8	56	

*K* = ($\frac{96}{108}-\frac{48.59}{108}$)/($\frac{108}{108}-\frac{48.59}{108}$) = 0.8; SD_(K)_ = 0.05; 95% CI = K ± Z_α/2_ SD_(K)_; K ± 1.96 (0.054) = 0.80 ± 0.10; Kappa (95% CI) = 0.80 (0.70, 0.90).
